# Study protocol for a randomised controlled trial of internet-based cognitive-behavioural therapy for obsessive-compulsive disorder

**DOI:** 10.1186/1471-244X-14-209

**Published:** 2014-07-25

**Authors:** Michael Kyrios, Maja Nedeljkovic, Richard Moulding, Britt Klein, David Austin, Denny Meyer, Claire Ahern

**Affiliations:** Swinburne University of Technology, Melbourne, VIC Australia; Centre for Mental Health and Wellbeing Research, Deakin University, Melbourne, VIC Australia; DVC-R Portfolio, School of Health Sciences, and the Collaborative Research Network, Federation University, Ballarat, VIC Australia; Centre for Mental Health Research, The Australian National University, Canberra, ACT Australia

**Keywords:** Mental health, Online intervention, Obsessive-compulsive disorder, OCD, Cognitive-behaviour therapy, CBT

## Abstract

**Background:**

Obsessive-Compulsive Disorder (OCD) is a common chronic psychiatric disorder that constitutes a leading cause of disability. Although Cognitive-Behaviour Therapy (CBT) has been shown to be an effective treatment for OCD, this specialised treatment is unavailable to many due to access issues and the social stigma associated with seeing a mental health specialist. Internet-based psychological treatments have shown to provide effective, accessible and affordable treatment for a range of anxiety disorders, and two Randomised Controlled Trials (RCTs) have demonstrated the efficacy and acceptability of internet-based CBT (iCBT) for OCD, as compared to waitlist or supportive therapy. Although these initial findings are promising, they do not isolate the specific effect of iCBT. This paper details the study protocol for the first randomised control trial evaluating the efficacy of therapist-assisted iCBT for OCD, as compared to a matched control intervention; internet-based therapist-assisted progressive relaxation training (iPRT). It will aim to examine whether therapist-assisted iCBT is an acceptable and efficacious treatment, and to examine how effectiveness is influenced by patient characteristics.

**Method/design:**

A randomised controlled trial using repeated measures with two arms (intervention and matched control) will be used to evaluate the efficacy and acceptability of iCBT for OCD. The RCT will randomise 212 Australian adults with a primary diagnosis of OCD into either the active intervention or control condition, for 12 weeks duration. Outcomes for participants in both study arms will be assessed at baseline and post-intervention. Participants in iCBT will be further assessed at six month follow-up, while participants in the control condition will be crossed over to receive the iCBT intervention and reassessed at post-intervention and six month follow-up. The primary outcome will be clinically significant change in obsessive-compulsive symptom scores.

**Discussion:**

This will be the first known therapist assisted internet-based trial of a comprehensive CBT treatment for OCD as compared to a matched control intervention. Demonstrating the efficacy of an internet-based treatment for OCD will allow the development of models of care for broad-based access to an evidence-based but complex treatment.

## Background

Obsessive-Compulsive Disorder (OCD) is a severe and incapacitating disorder associated with intense anxiety, high degrees of psychiatric comorbidity, and significant health and social costs [1]. The disorder is recognised as a leading cause of disability [2] with levels of impairment similar to that of patients with schizophrenia [1]. OCD is characterised by the presence of obsessions and/or compulsions [3]. Obsessions are unwanted ego-dystonic thoughts, images or urges that are recurrent, persistent and intrusive, and that lead to marked distress. Compulsions are repetitive, rigid intentional behaviours or mental acts performed to reduce distress that follows an obsession, or to avoid potential danger [3]. OCD is a highly complex and heterogenous disorder with differential treatment outcomes for its various presentations [4].

Cognitive models of OCD have prompted recent advances in its psychological treatment. Central to the model is the understanding that normal unwanted intrusions (i.e., thoughts, images, urges) form the basis of obsessions [5]. Unwanted intrusions are universal in the experiences of the general population, and normal intrusions are indistinguishable from clinical obsessions. However, misappraisals of intrusions as personally significant, meaningful or dangerous, lead to anxiety or discomfort, and provoke maladaptive responses (e.g., compulsions) to alleviate distress [6]. Although these compulsions reduce discomfort in the short term, they are negatively reinforced and ultimately serve to maintain the misappraisals. Attempts at neutralising the unwanted intrusions paradoxically increase the intrusion’s salience, difficulty of dismissal, and intensity. Misinterpretation and neutralisation or compulsive actions thus maintain the occurrence of obsessions and further strengthen maladaptive beliefs.

### Cognitive behaviour therapy for OCD

In targeting such processes in cognitive-behaviour therapy (CBT), patients monitor thoughts, evaluate evidence for maladaptive beliefs and appraisals, and develop and practice more productive appraisals to counter cognitive biases. Behavioural experiments and other cognitive techniques that help test alternative interpretations of intrusions are a key strategy in the treatment of OCD [7], but these techniques are somewhat more specialised and less accessible than the more widely known exposure-based strategies [8]. Exposure and Response Prevention (ERP) is an evidence-based central behavioural technique requiring individuals to face situations that induce distress while refraining from engaging in compulsive rituals [9, 10]. In doing so, ERP leads to the extinction of avoidance and anxiety responses. Anxiety management and coping strategies such as relaxation techniques also have some credibility and acceptability amongst patients as they are useful in decreasing anxiety; however, they are generally no longer centrally utilised in CBT programs as the evidence for their efficacy is inconsistent, although they may be useful when the person has extreme anxiety responses [10].

Current clinical guidelines recommend CBT as a first line treatment for OCD [11]. CBT programs are typically 12 – 24 hours duration, with drop-out rates of around 25% and recovery rates at post-treatment of 50% to 75% [10]. Systematic reviews and meta-analyses support that CBT programs are effective in reducing OCD symptoms, with effect sizes ranging from 0.8 to 1.24 from pre to post treatment [10–15]. Although effective treatments exist, only a small percentage (<10%) of individuals with OCD receive CBT [16–18]. The mean duration between onset of first OCD symptoms and presentation for treatment is around seven years [19, 20]. Individuals may go undiagnosed and untreated for many years due to a failure of health professionals to recognise OCD [21] and to intense feelings of embarrassment and guilt motivating patients to not disclose their experiences [16, 22]. For those that do present for help, access to evidence-based treatment is poor; a shortage of appropriately qualified professionals (especially in geographically remote areas), long waitlists, and individuals’ financial constraints mean that specialised evidence-based treatment is often unavailable [8].

### Use of internet in mental health treatment to increase accessibility

Internet access is increasing across the world [23]. In Australia, the proportion of households with Internet access at home in 2012–13 was 83%, an increase from 79% in 2010–11 [24]. It is not surprising that the majority of people (77%) seek out mental health information from the internet [25]. Hence, internet interventions represent a unique opportunity to deliver evidence-based mental health treatment to large segments of the population, who might otherwise be unable to access such treatment [26, 27]. Internet-based therapy has distinct potential advantages. It allows dissemination of standardised yet personalised treatments, which may include contact with therapists over e-mail or other modes of remote communication (e.g., telephone, video-chat). This is particularly beneficial for patients in geographically isolated regions, who may not have access to specialised mental health professionals. In contrast to face-to-face services, internet-based treatments can be accessed 24 hours a day, 7 days a week, without affecting efficiency while increasing personal convenience and being generally a lower cost option than face-to-face equivalents [28]. A further advantage of internet-based treatments is that they can be easily utilised in conjunction with other treatments, inclusive of pharmacotherapy and face-to-face supports such as supportive psychotherapy, general practice review and other psychological interventions. Particularly important for OCD, internet-based therapies allow anonymity, and in some cohorts experiencing stigma, individuals feel more comfortable with using technology than discussing their concerns in person [29–31].

Internet-based mental health interventions have been established as an effective model for the provision of CBT, without the need for intensive therapist involvement, for a variety of disorders and/or symptoms, including depression [32], panic disorder [26, 33, 34], post-traumatic stress disorder [35], and specific phobias [36]. Reviews of randomised controlled trials (RCTs) of internet interventions with therapist assistance for depressive and anxiety disorders have found reductions in symptoms, relevant cognitions and improvement in patients’ mental health literacy [37–39]. Interventions with therapist-assistance generally show larger effect sizes and lower attrition rates than self-help programs [38], while the amount and nature of the therapists’ experience has not been found to greatly influence the clinical outcome of internet therapy [40].

### Internet-based interventions for OCD

Although a relatively new area of investigation, a few open trials have been reported [41, 42], with only two RCTs demonstrating the efficacy and acceptability of internet-based CBT (iCBT) for OCD. The first and largest RCT to date by Andersson et al. [43] comprised 101 individuals with a primary diagnosis of OCD who were not currently receiving counseling, and had no recent history of CBT (2 years) or changes to medication (2 months). Eligible participants were randomly allocated to receive 10-week therapist guided iCBT or a 10-week control condition (online supportive therapy). The iCBT condition was associated with a significant reduction in OCD symptoms (*d* = 1.55) as measured by the Yale Brown Obsessive Compulsive Scale (YBOCS; [44]), compared to a medium within-group effect size (*d* = 0.47) for the Control Group. Additionally, there was a large between between-group effect size (*d* = 1.12) on the YBOCS, and only 6% participants in the control condition met criteria for clinically significant change, compared with 60% in iCBT. The results of iCBT were maintained at 4-month follow up. Although therapist contact in the control group was significantly lower than iCBT (129 minutes versus 17 minutes for iCBT and control, respectively), the between groups difference remained significant after therapist contact was statistically controlled. Nevertheless the specific effect of iCBT is unclear, as the control condition did not provide intervention elements that were experienced as similar to the active treatment (e.g., online self-help information, downloadable audio files, worksheets and homework).

Similar results were found in a smaller RCT by Wootton et al. [45] which involved 56 individuals with OCD who were not currently receiving CBT and had not had any changes to medication within the previous month. The authors found that 8-week therapist guided iCBT (N = 17) and therapist guided bibliotherapy (bCBT; N = 20) were effective compared to a waitlist control condition (N = 19; between-group effect sizes of *d* = 1.57 and 1.40, respectively). The experimental groups were associated with a 47% (bCBT) and 39% (iCBT) reduction in OCD symptoms scores, which were maintained at 3-month follow-up, with a mean total therapist time of approximately 103 minutes for bCBT and 89 minutes for iCBT. The within group effect sizes for the experimental groups were larger than those obtained by self-help versions of the same program [41], suggesting that therapist assisted iCBT had stronger treatment effects. The same research group demonstrated similar efficacy in an earlier open trial of therapist-guided iCBT [46], which was rated as highly acceptable by participants despite their receiving an average of only 86 minutes of therapist contact.

These studies demonstrate that large effect sizes, which were comparable to those obtained in face-to-face therapy [47, 48], can be obtained with minimal therapist contact, although follow-up periods have been relatively shorter than in other outcome studies for OCD that usually report 6-to-12 month follow-up data. Given that a standard course of face-to-face CBT would be around 6–12 sessions of one hour, online therapies for OCD are shown to be not only efficacious, but also cost effective. These economic, acceptability and efficacy considerations make it imperative that there is further examination into the utility of online therapy for OCD. In particular, RCTs that compare iCBT to a matched control are required to help isolate whether therapeutic outcomes are due to CBT and are independent of other intervention elements such as treatment credibility, therapist attention, therapeutic alliance, time, and use of an online modality and associated therapeutic tools (e.g., downloadable audiovisual components, homework tasks, etc.). Given the complexity and heterogeneity in presentations of OCD, therapist-assisted programs are best suited to help tailor intervention programs to individual client needs, and to promote treatment engagement and adherence.

### The current study

This paper details the study protocol for an RCT evaluating the efficacy of therapist-assisted iCBT for OCD. The primary aim is to examine whether therapist-assisted iCBT is an acceptable and efficacious treatment, as compared to an analogous active control, for patients who can choose to continue with their usual treatment. Treatment as usual is particularly well-suited to answer the practical question of whether introducing the new treatment could improve outcomes over and above the current state of practice. A further aim is to examine how effectiveness is influenced by patient and other characteristics.

### Primary outcome

The primary outcomes will be clinically significant change in obsessive-compulsive symptom scores and diagnostic status. Our primary hypothesis is that individuals in the iCBT and matched control groups will experience reduction in the proportion of diagnosable OCD and significant alleviation of OCD symptoms from pre to post-intervention, with significantly greater improvements in iCBT. It is further anticipated that iCBT outcomes would be maintained at 6-to-12 month follow-up.

### Secondary outcome

Secondary outcomes will be general mental health (depression and anxiety), psychological variables (cognitions related to OCD, self-efficacy), and psychosocial variables (quality of life, functional impairment). We expect that individuals randomised to receive iCBT, compared to matched controls, will demonstrate significantly greater improvements in secondary outcome measures from pre- to post-intervention. We also expect iCBT to be associated with lower service utilisation than the matched control condition.

Demographic and clinical variables, treatment credibility and expectations of treatment, treatment acceptability, working alliance between patient and therapist, and attitudes towards homework tasks will also be assessed. We expect that: a) participants will rate iCBT as more credible and acceptable than the matched control group, but that there will be no differences between groups on working alliance and attitudes towards homework; and b) outcomes will be positively associated with homework completion, but other predictors (e.g. education, gender, age, degree to which other treatments were undertaken) are not expected to contribute to outcomes. Finally, the present study proposes to explore how individual patient characteristics may influence therapeutic outcomes (e.g., demographic variables, functional impairment, treatment readiness, personality, diagnostic/symptom severity, specific symptom presentation, levels of comorbidity, expectancies of treatment credibility).

## Methods/design

**Figure 1:**
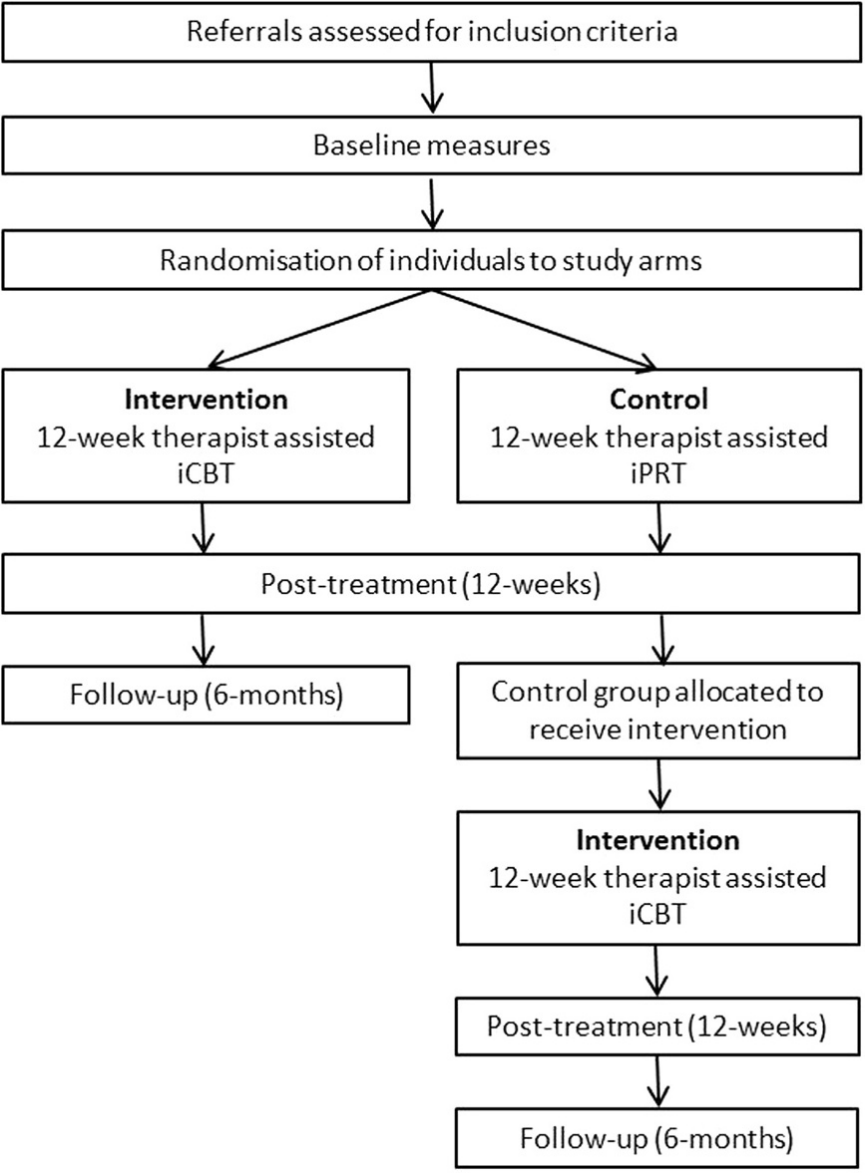
**CONSORT flowchart.**

The study will evaluate the acceptability and efficacy of iCBT compared to a matched control condition. The RCT will randomise 212 individuals who fulfil inclusion criteria into either the active treatment or control condition, for 12 weeks duration (Figure 1). Outcomes for participants in both groups will be assessed at baseline (Time = 0) and post-intervention (Time = 12 weeks). Participants in iCBT will be further assessed at follow-up (Time = 6-to-12 months), while participants in the control will be crossed over to receive the intervention and assessed at post-intervention (Time = 24 weeks) and follow-up (Time = 9 months).

### Ethics approval

The present study proposal has been subject to peer review and approval by the National Health and Medical Research Council (Project Grant Application 620506). Ethics approval has been obtained from the Human Research Ethics Committees from Swinburne University of Technology (No. 2010/104).

### Participants

Recruitment will occur in all states and territories of Australia, and will be open to all Australian residents aged 18 years and over. Two hundred and twelve individuals with a primary diagnosis of OCD, as indicated by structured clinical interview, will be recruited through widespread media coverage, internet advertising (e.g., Facebook), web search engines (e.g., Google) and referrals from other online treatment services (e.g., Anxiety Online, Mental Health Online; [49]). Consumer organisations and mental health services/professionals (e.g., university psychology clinics, divisions of general practice, psychiatric facilities, private psychologists and psychiatrists) will be approached to promote the program and invited to make referrals.

### Eligibility criteria

All interested participants will be required to complete a telephone interview and fulfil Diagnostic and Statistical Manual for Mental Disorders (DSM-IV) criteria for OCD on the Structured Clinical Interview for DSM-IV (SCID) [50]. All participants will be required to have sufficient English to complete treatment and assessments without translation, provide multiple contact details, be able and willing to access internet and email, and consent to follow up assessments. Comorbid diagnosis of other disorders will be permitted where OCD is the primary diagnosis, and the non-OCD diagnosis is not in the extremely severe range. Exclusion criteria include meeting criteria for current psychosis, substance abuse, head injury or neurological disorder, and active suicidal ideation. Checks with participants about other treatments received will be maintained during the study.

### Sample size calculation

Given previous research showing large effects for online CBT for OCD [43, 45], we expect a large effect for iCBT (i.e., 33% achieving clinically significant change in YBOCS scores; Cohen’s d of approximately 1.0). Progressive relaxation training (PRT) will be the active attention control [51]. PRT has demonstrated inconsistent effectiveness in treating OCD with studies indicating low [52] and high efficacy [53]. Given this, we anticipate a moderate effect size for this group (i.e., 15% achieving clinically significant change in YBOCS scores; Cohen’s d approximately 0.5). Therefore, 88 individuals per intervention group will be sufficient to detect a difference in effects between iCBT and control at post-intervention, assuming significance is set at 5% (α = 0.05) power at 80% and a standard deviation of 5 for the distribution of mean change. We include an additional 20% (N = 18 per group) to allow for attrition and potential effects of various extraneous influences, such as protocol adherence; 106 participants per group will be required (total = 212).

### Participant recruitment procedure

Participants will make initial contact via email or telephone. Those who express an interest will be emailed a Participant Information and Consent Form. Consenting participants will be asked to provide contact details to arrange the telephone interview (to assess eligibility and collect baseline measures). At the telephone interview, those participants not meeting criteria will be given information about alternative treatment and relevant local services and internet sites. If participation criteria are met, consent will be sought for further baseline assessment, treatment and follow-up.

### Randomisation procedure

In accordance with CONSORT guidelines [54], randomisation will occur after participants have completed their baseline measures. Although there is a generally equal gender prevalence of OCD in adults, there are inconsistent reports of gender differences in service utilization rates [55]. Therefore, stratified randomisation will be used in order to minimize the differences in gender distributions across and within groups. This will be done via computer software and entered by an independent researcher. Participants will be allocated to a therapist and given immediate access to their online condition (intervention or control), and participants in the control condition given access to the intervention after completing the post-intervention assessment. The CONSORT diagram of the study design is shown in Figure 1.

### Study arms

Throughout the course of the study, participants from both the intervention and control groups will continue to receive treatment as usual (TAU). Structured checks with participants about other treatments will be maintained at all assessment points with assessors blind to condition. Both groups receive an online program consisting of 12 modules over 12-weeks. Participants will be encouraged to complete one module per week for the duration of treatment. Both groups will receive online information, weekly homework tasks, downloadable worksheets and audio files. All participants will receive weekly email support from a remote therapist who will monitor progress, review homework, provide support and encouragement, and give assistance in tailoring the treatment to the participant’s individual problem, in line with the online content of their allocated condition. The communication will be restricted to one therapist email per week, although users will be able to email their therapists as often as they wish. Treatment will be via automated programs. Assessors and therapists will be licenced psychologists, or supervised students enrolled in a Masters or Professional Doctorate in Clinical Psychology. Therapy provision and assessment will be conducted with independent assessors. All therapists will be trained and supervised on a fortnightly review basis by a Clinical Psychologist experienced in the treatment of OCD. Email therapy sessions will be saved and rated by independent assessors to assess treatment fidelity, and interrater reliability assessments will be conducted to confirm diagnosis.

### Intervention: Therapist-assisted internet-based cognitive behavioural therapy (iCBT)

The intervention is a 12-week internet-based cognitive behavioural therapy. Modules 1–3 provide information about OCD, anxiety, and an introduction to CBT, along with anxiety and depression management strategies (e.g., relaxation, activity scheduling). In Modules 4–6, Exposure and Response Prevention (ERP) strategies are introduced through the construction of fear hierarchies. These teach the participant to plan and implement an ERP program suited to their individual needs. Due to the heterogeneity of the disorder, participants are provided with a range of examples to correspond to various presentations. Modules 7–9 include cognitive therapy techniques (e.g., cognitive restructuring) targeting OCD specific cognitive styles (such as inflated responsibility/overestimation of threat, importance/control of thoughts). As part of this, cognitive misappraisals are identified and alternatives developed and practiced; and more advanced ERP, integrating cognitive strategies is undertaken. Modules 10–12 include relapse preventions strategies (e.g., problem solving, risk identification, contingency management, and mindfulness techniques). The importance of daily practice is emphasised and discussions focus on enablers of, and barriers to, maintenance of CBT.

### Control: Therapist-assisted internet-based progressive relaxation training (iPRT)

The matched control condition is an internet-based treatment for anxiety adapted from Bernstein et al. [56]. Participants initially receive basic information about OCD, the relationship of OCD to anxiety, and the use of relaxation to manage anxiety. Individuals are then taught to relax specific muscle groups while paying attention to the feelings associated with both the tensed and relaxed stages. The program teaches the individual to achieve a state of deep relaxation in increasingly shorter periods and to control excess tension in stress-inducing situations. Participants work through the sequential tensing and releasing of 16 muscle groups (Modules 1 to 3). These groups are reduced to seven (Modules 4 and 5) and to four (Modules 6 to 7). The tension component is then withdrawn (Modules 8 to 11) in what is called “relaxation through recall”, and the final stage (Module 12) consists of a mental summary of the previously learned techniques. Participants will have access to downloadable audiovisual and written material to guide their progressive relaxation training. Although relaxation training has previously not been considered effective for OCD [52], recent research supports that progressive relaxation can be effective [53]. Considering that the program requires more than attention and engagement, the term ‘matched control’ was deemed a more appropriate descriptor than ‘active attention placebo’.

### Measures

Outcomes will be assessed via clinician-rated telephone interview at baseline, post-intervention and follow-up. Throughout the intervention and control, participants will self-report secondary outcomes through an online questionnaire embedded within Modules 1, 5, 9 and 12 of each condition. Instruments used to collect data from participants, including primary and secondary outcomes, are listed in Table 1.

**Table 1:** **Instruments used for data collection**

Measurement tools/questions		Time point			
Clinician administered (telephone)		Baseline	Post-treat		Follow-up
Demographics	Age, gender, postcode, family characteristics, religion, education, general medical history	x			
Current treatment	Current physiological & psychological treatment	x	x		x
Diagnosis	Structured Clinical Interview for DSM-IV Axis I Disorders - Clinician Version (SCID-IV) [50]	x	x		x
OCD severity	Yale-Brown Obsessive-Compulsive Scale (YBOCS) [44]	x	x		x
	Obsessive-Compulsive Inventory (OCI) [57]	x	x		x
	Clinical Global Improvement Scale (CGI) [58]	x	x		x
Mental health	Hamilton Rating Scale for Depression (HRSD) [59]	x	x		X
	Hamilton Rating Scale for Anxiety (HRSA) [60]	x	x		x
		**Modules**			
**Self-report (online)**		**1**	**5**	**9**	**12**
OCD measures	Yale-Brown Obsessive-Compulsive Scale (YBOCS) [44]	x			x
	Obsessive Compulsive Inventory (OCI) [61]	x	x	x	x
	Revised Obsessional Beliefs Questionnaire (OBQ) [62]	x	x	x	x
Psychosocial	Depression, Anxiety, Stress Scale (DASS) [63]	x	x	x	x
	Centre for Epidemiologic Studies Depression Scale (CES-D) [64]	x			
	General Self-Efficacy Scale (GSES) [65]	x			
	Australian Quality of Life Scale (AQoLS) [66]	x			x
	Brief Disability Questionnaire (BDQ) [67]	x			x
	Service Utilisation & Loss of Role Questionnaire (SULRQ) [68]	x			x
Treatment measures	Credibility/Expectancy Questionnaire (CEQ) [69]	x			
	Current Treatments Measure [70]	x			x
	Treatment Evaluation Inventory (TEI) [71]	x			x
	Working Alliance Questionnaire (WAQ) [72]	x			x
	Homework Rating Scale (HSR-II) [73]	x	x	x	x

### Data analysis

Exploratory and confirmatory factor analysis will be used to check the psychometric properties of the various scales. Where appropriate, scales will be adjusted and further validation performed to ensure that the resulting scales have internal validity and reliability. The primary analyses will utilise a within and between groups design with two time points (baseline and post-intervention) and two groups (iCBT versus control). Repeated measures analyses will be conducted on follow-up outcomes for the group initially assigned to the iCBT condition. *Clinically significant change* will be based on a combination of reliable reduction in YBOCS scores and post-treatment YBOCS outside the clinical range [74].

Despite randomisation, there are advantages in accounting for potential biases in assignment to treatments [32]. Similarity of baseline characteristics of intervention and control participants will be assessed using appropriate summary statistics and use of multi-level modeling will allow comparison of the iCBT and control conditions in terms of pre-/post changes in primary and secondary outcomes, while controlling for relevant confounds. An intention to treat and completer analyses, and then a longitudinal hierarchical linear model analyses, will be conducted to avoid the need to impute missing data, and baseline data will be examined to analyse probability of attrition bias.

### Trial status

The trial is currently in the data collection phase. Recruitment to the study commenced in 2010. To date, from over 1300 referrals that expressed interest in the study either online or via telephone, 197 participants have provided consent and completed the pre-intervention interview via telephone. Applicants who met inclusion criteria (n = 179) completed baseline assessments and were randomised into the intervention (n = 91) and control (n = 88) conditions. It is anticipated that full post-intervention and follow up data will be completed by December, 2014.

## Discussion

Internet access is increasingly available, and reliance on the internet for healthcare information and treatments will increase with improved infrastructure. This paper provides a comprehensive description of the methodology used to implement, disseminate and evaluate an online cognitive-behavioural therapy program for OCD. Although other treatments exist and RCTs have been conducted [51, 75], evaluations using study designs controlling for expectancy/attention-placebo effects are still required. This will be the first known therapist assisted internet-based trial of a comprehensive CBT treatment (iCBT) for OCD as compared to a matched control (iPRT) for patients who have a choice of undertaking other concurrent treatment. The added advantage of this design is that it encompasses a more representative cohort of patients undertaking internet-based psychological treatment for OCD by not excluding those undertaking other concurrent treatments or those with a wide range of comorbidity. The iCBT program will provide necessary assistance by remote contact with a suitably qualified mental health professional, providing greater access to a specialised OCD service, particularly for those from rural and remote areas, whilst also encouraging active patient self-management. Hence, this project serves as a contemporary model for greater access to flexible, innovative, affordable and evidence-based psychological treatment for OCD. Demonstrating the efficacy of such a program will facilitate its integration into clinical practice and the development of models of care for the management of OCD.

## Abbreviations

NHMRC: National Health and Medical Research Council
OCD: Obsessive-compulsive disorder
CBT: Cognitive-behaviour therapy
RCT: Randomised controlled trial
iCBT: Internet-based cognitive-behaviour therapy
iPRT: Internet-based progressive relaxation training
ANZCTR: Australian New Zealand Clinical Trials Registry
ERP: Exposure and response prevention
bCBT: Bibliotherapy cognitive-behavioural therapy
DSM-IV: Diagnostic and statistical manual for mental disorders
PRT: Progressive Relaxation Training.

## References

[1] Torresan RC, Smaira SI, Ramos-Cerqueira ATDA, Torres AR (2008). Quality of life in obsessive-compulsive disorder: A review. Qualidade de vida no transtorno obsessivo-compulsivo: Uma revisão.

[2] Ayuso-Mateos JL (2006). Global Burden of Obsessive-compulsive Disorder in the Year 2000.

[3] American Psychiatric Association [APA] (2000). Diagnostic and Statistical Manual - Text Revision (DSM-IV-TR).

[4] McKay D, Abramowitz JS, Calamari JE, Kyrios M, Radomsky A, Sookman D, Taylor S, Wilhelm S (2004). A critical evaluation of obsessive-compulsive disorder subtypes: symptoms versus mechanisms. Clin Psychol Rev.

[5] Rachman S (1997). A cognitive theory of obsessions. Behav Res Ther.

[6] Frost R, Steketee G, Amir N, Bouvard M, Carmin C, Clark DA, Cottraux J, Eisen J, Emmelkamp P, Foa E, Obsessive Compulsive Cognitions Working Group (1997). Cognitive assessment of obsessive-compulsive disorder. Behav Res Ther.

[7] Freeston MH, Rhéaume J, Ladouceur R (1996). Correcting faulty appraisals of obsessional thoughts. Behav Res Ther.

[8] Kyrios M, Moulding R, Jones B (2010). Obsessive compulsive disorder: Integration of cognitive-behaviour therapy and clinical psychology care into the primary care context. Aust J Prim Health.

[9] Kyrios M, Menzies R, Chichester DSP (2003). Exposure and response prevention in the treatment of Obsessive–Compulsive Disorder. Obsessive-Compulsive Disorder: Theory, Research and Treatment.

[10] Abramowitz JS, Taylor S, McKay D (2005). Potentials and limitations of cognitive treatments for obsessive-compulsive disorder. Cogn Behav Ther.

[11] National Institute for Health and Clinical Excellence (NICE) (2006). Core Interventions in the Treatment of OCD and BDD.

[12] Ponniah K, Magiati I, Hollon SD (2013). An update on the efficacy of psychological treatments for obsessive-compulsive disorder in adults. J Obsessive-Compuls Relat Disord.

[13] Jónsson H, Hougaard E (2009). Group cognitive behavioural therapy for obsessive-compulsive disorder: A systematic review and meta-analysis. Acta Psychiatr Scand.

[14] Olatunji BO, Davis ML, Powers MB, Smits JAJ (2013). Cognitive-behavioral therapy for obsessive-compulsive disorder: A meta-analysis of treatment outcome and moderators. J Psychiatr Res.

[15] Ougrin D (2011). Efficacy of exposure versus cognitive therapy in anxiety disorders: Systematic review and meta-analysis. BMC Psychiatry.

[16] Torres AR, Prince MJ, Bebbington PE, Bhugra DK, Brugha TS, Farrell M, Jenkins R, Lewis G, Meltzer H, Singleton N (2007). Treatment seeking by individuals with obsessive-compulsive disorder from the british psychiatric morbidity survey of 2000. Psychiatr Serv.

[17] Blanco C, Olfson M, Stein DJ, Simpson HB, Gameroff MJ, Narrow WH (2006). Treatment of obsessive-compulsive disorder by U.S. psychiatrists. J Clin Psychiatry.

[18] Schwartz C, Schlegl S, Kuelz AK, Voderholzer U (2013). Treatment-seeking in OCD community cases and psychological treatment actually provided to treatment-seeking patients: A systematic review. J Obsessive Compuls Relat Disord.

[19] Feinstein SB, Fallon BA, Petkova E, Liebowitz MR (2003). Item-by-item factor analysis of the Yale-Brown Obsessive Compulsive Scale Symptom Checklist. J Neuropsychiatry Clin Neurosci.

[20] Rasmussen SA, Tsuang MT (1986). Clinical characteristics and family history in DSM-III obsessive-compulsive disorder. Am J Psychiatry.

[21] Wahl K, Kordon A, Kuelz KA, Voderholzer U, Hohagen F, Zurowski B (2010). Obsessive-Compulsive Disorder (OCD) is still an unrecognised disorder: A study on the recognition of OCD in psychiatric outpatients. Eur Psychiatry.

[22] Marques L, LeBlanc NJ, Wegarden HM, Timpano KR, Jenike M, Wilhelm S (2010). Barriers to treatment and service utilization in an internet sample of individuals with obsessive-compulsive symptoms. Depress Anxiety.

[23] (BDT) TDB (2013). Yearbook of Statistics - Telecommunication/ICT Indicators - 2003–2012.

[24] Statistics ABo (2014). Multipurpose Household Survey (MPHS) for 2012–13.

[25] Burns JM, Davenport TA, Durkin LA, Luscombe GM, Hickie IB (2010). The internet as a setting for mental health service utilisation by young people. Med J Aust.

[26] Carlbring P, Ekselius L, Andersson G (2003). Treatment of panic disorder via the Internet: a randomized trial of CBT vs. applied relaxation. J Behav Ther Exp Psychiatry.

[27] Parslow RA, Jorm AF (2000). Who uses mental health services in Australia? An analysis of data from the national survey of mental health and wellbeing. Aust N Z J Psychiatry.

[28] Andersson G, Titov N (2014). Advantages and limitations of Internet-based interventions for common mental disorders. World Psychiatry.

[29] Wallach HS, Safir MP, Bar-Zvi M (2009). Virtual reality cognitive behavior therapy for public speaking anxiety: A randomized clinical trial. Behav Modif.

[30] Wilson JAB, Onorati K, Mishkind M, Reger MA, Gahm GA (2008). Soldier attitudes about technology-based approaches to mental health care. Cyberpsychol Behav.

[31] Zinzow HM, Britt TW, McFadden AC, Burnette CM, Gillispie S (2012). Connecting active duty and returning veterans to mental health treatment: Interventions and treatment adaptations that may reduce barriers to care. Clin Psychol Rev.

[32] Christensen H, Griffiths KM, Jorm AF (2004). Delivering interventions for depression by using the internet: Randomised controlled trial. Br Med J.

[33] Kenardy J, McCafferty K, Rosa V (2003). Internet-delivered indicated prevention for anxiety disorders: A randomized controlled trial. Behav Cogn Psychother.

[34] Klein B, Richards JC (2001). A brief internet-based treatment for panic disorder. Behav Cogn Psychother.

[35] Lange A, van de Ven JP, Schrieken B (2003). Interapy: Treatment of post-traumatic stress via the internet. Cogn Behav Ther.

[36] Kenwright M, Marks IM (2004). Computer-aided self-help for phobia/panic via internet at home: A pilot study. Br J Psychiatry.

[37] Griffiths KM, Farrer L, Christensen H (2010). The efficacy of internet interventions for depression and anxiety disorders: A review of randomised controlled trials. Med J Aust.

[38] Spek V, Cuijpers P, Nyklicek I, Riper H, Keyzer J, Pop V (2007). Internet-based cognitive behaviour therapy for symptoms of depression and anxiety: a meta-analysis. Psychol Med.

[39] Christensen H, Batterham P, Calear A (2014). Online interventions for anxiety disorders. Curr Opin Psychiatry.

[40] Andersson G, Carlbring P, Furmark T (2012). Therapist experience and knowledge acquisition in internet-delivered CBT for social anxiety disorder: A randomized controlled trial. PLoS ONE.

[41] Wootton BM, Dear BF, Johnston L, Terides MD, Titov N (2014). Self-guided internet administered treatment for obsessive-compulsive disorder: Results from two open trials. J Obsessive Compuls Relat Disord.

[42] Andersson E, Ljótsson B, Hedman E, Kaldo V, Paxling B, Andersson G, Lindefors N, Rück C (2011). Internet-based cognitive behavior therapy for obsessive compulsive disorder: A pilot study. BMC Psychiatry.

[43] Andersson E, Enander J, Andrén P, Hedman E, Ljótsson B, Hursti T, Bergström J, Kaldo V, Lindefors N, Andersson G, Rück C (2012). Internet-based cognitive behaviour therapy for obsessive-compulsive disorder: A randomized controlled trial. Psychol Med.

[44] Goodman WK, Price LH, Rasmussen SA, Mazure C, Fleischmann RL, Hill CL, Heninger GR, Charney DS (1989). The Yale-Brown Obsessive Compulsive Scale. I. Development, use, and reliability. Arch Gen Psychiatry.

[45] Wootton BM, Dear BF, Johnston L, Terides MD, Titov N (2013). Remote treatment of obsessive-compulsive disorder: A randomized controlled trial. J Obsessive Compuls Relat Disord.

[46] Wootton BM, Titov N, Dear BF, Spence J, Andrews G, Johnston L, Solley K (2011). An Internet administered treatment program for obsessive-compulsive disorder: a feasibility study. J Anxiety Disord.

[47] Kobak KA, Greist JH, Jefferson JW, Katzelnick DJ, Henk HJ (1998). Behavioral versus pharmacological treatments of obsessive compulsive disorder: a meta-analysis. Psychopharmacology (Berl).

[48] Rosa-Alcazar AI, Sanchez-Meca J, Gomez-Conesa A, Marin-Martinez F (2008). Psychological treatment of obsessive-compulsive disorder: a meta-analysis. Clin Psychol Rev.

[49] Klein B, Meyer D, Austin DW, Kyrios M (2011). Anxiety online-A virtual clinic: Preliminary outcomes following completion of five fully automated treatment programs for anxiety disorders and symptoms. J Med Internet Res.

[50] First M, Spitzer R, Gibbon M, Williams J (1996). Structured Clinical Interview for DSM-IV Axis I Disorders, Clinician Version (SCID-CV).

[51] Marks I, Cavanagh K (2009). Computer-aided psychological treatments: evolving issues. Annu Rev Clin Psychol.

[52] Lindsay M, Crino R, Andrews G (1997). Controlled trial of exposure and response prevention in obsessive- compulsive disorder. Br J Psychiatry.

[53] Twohig MP, Hayes SC, Plumb JC, Pruitt LD, Collins AB, Hazlett-Stevens H, Woidneck MR (2010). A randomized clinical trial of acceptance and commitment therapy versus progressive relaxation training for obsessive-compulsive disorder. J Consult Clin Psychol.

[54] Moher D, Schulz K, Arltman D (2001). The CONSORT statement: Revised recommendations for improving the quality of reports of parallel group randomized trials. BMC Med Res Methodol.

[55] Johnson EM, Coles ME (2013). Failure and delay in treatment-seeking across anxiety disorders. Community Ment Health J.

[56] Bernstein D, Borkovec T, Hazlett-Stevens H (2000). New Directions in Progressive Relaxation Training: A Guidebook for Helping Professionals.

[57] Foa E, Huppert JD, Leiberg S, Langner R, Kichic R, Hajcak G, Salkovskis P (2002). The obsessive-compulsive inventory: Development and validation of a short version. Psychol Assess.

[58] Guy W (1976). ECDEU Assessment Manual for Psychopharmacology —Revised.

[59] Hamilton M (1960). A rating scale for depression. J Neurol Neurosurg Psychiatry.

[60] Hamilton M (1959). The assessment of anxiety states by rating. Br J Med Psychol.

[61] Foa EB, Kozak MJ, Salkovskis PM, Coles ME, Amir N (1998). **The validation of a new obsessive-compulsive disorder scale: The Obsessive-Compulsive Inventory.**. Psychological Assessment.

[62] Steketee G, Frost R, Bhar S, Bouvard M, Calamari J, Carmin C, Clark DA, Cottraux J, Emmelkamp P, Forrester E, Obsessive Compulsive Cognitions Working Group (2003). Psychometric validation of the Obsessive Beliefs Questionnaire and the Interpretation of Intrusions Inventory: Part I. Behav Res Ther.

[63] Lovibond S, Lovibond P (1995). Manual for the Depression Anxiety Stress Scales.

[64] Lewinsohn P, Seeley J, Roberts R, Allen N (1997). Center for Epidemiological Studies-Depression Scale (CES-D) as a screening instrument for depression among community-residing older adults. Psychol Aging.

[65] Jerusalem M, Schwarzer R, Schwarzer R (1992). Self-efficacy as a resource factor in stress appraisal processes. Self-efficacy: Thought Control of Action.

[66] Hawthorne G, Richardson J, Osborne R, McNeil H (1997). The Australian Quality Of Life (AQoL) Instrument.

[67] Von Korff M, Ustun TB, Ormel J, Kaplan I, Simon GE (1996). Self-report disability in an international primary care study of psychological illness. J Clin Epidemiol.

[68] Nedeljkovic M (2011). Protocol for an Economic Evaluation of an Internet-based Psychological Intervention for Obsessive-Compulsive Disorder: Conducting an Economic Evaluation Alongside a Randomised Controlled Trial. Masters Thesis.

[69] Borkovec TD, Nau SD (1972). Credibility of analogue therapy rationales. J Behav Ther Exp Psychiatry.

[70] Gunn JM, Gilchrist GP, Chondros P, Ramp M, Hegarty KL, Blashki GA, Pond DC, Kyrios M, Herrman HE (2008). Who is identified when screening for depression is undertaken in general practice? Baseline findings from the Diagnosis, Management and Outcomes of Depression in Primary Care (diamond) longitudinal study. Med J Aust.

[71] Kazdin AE, French NH, Sherick RB (1981). Acceptability of alternative treatments for children: Evaluations by inpatient children, parents, and staff. J Consult Clin Psychol.

[72] Munder T, Wilmers F, Leonhart R, Linster HW, Barth J (2010). Working alliance inventory-short revised (WAI-SR): Psychometric properties in outpatients and inpatients. Clin Psychol Psychother.

[73] Kazantzis N, Deane F, Ronan K, Kazantzis N, Deane F, Ronan K, L'Abate L (2005). Assessment of homework completion. Using Homework Assignments in Cognitive Behavior Therapy.

[74] Jacobson NS, Truax P (1991). Clinical significance: a statistical approach to defining meaningful change in psychotherapy research. J Consult Clin Psychol.

[75] Marks IM, Cuijpers P, Cavanagh K, Van Straten A, Gega L, Andersson G (2009). Meta-analysis of computer-aided psychotherapy: Problems and partial solutions. Cogn Behav Ther.

[CR76] The pre-publication history for this paper can be accessed here:http://www.biomedcentral.com/1471-244X/14/209/prepub

